# “Okay in theory”: a qualitative study of safer sleep advice in families with infants at risk and families reporting risky sleep practices

**DOI:** 10.1136/bmjpo-2025-003620

**Published:** 2025-07-24

**Authors:** Becky Lambert, Alice-Amber Keegan, Jenny Ingram, Peter S Blair, Peter J Fleming, Anna Pease

**Affiliations:** 1 Centre for Academic Child Health, Bristol Medical School, University of Bristol, Bristol, UK

**Keywords:** Child Health, Health Policy, Infant, Psychology, Qualitative research

## Abstract

**ABSTRACT:**

**Background:**

Safer sleep messages have reduced the rates of sudden unexpected deaths in infancy by 90% in England since 1990. However, deaths continue, mainly in deprived families or where unsafe sleep practices remain common. Understanding the factors influencing these decisions can help further reduce these deaths. This study explores what influences infant sleep-related care practices among caregivers of at-risk infants and those who report non-adherence to safer sleep advice.

**Methods:**

A previously collected survey of infant care practices allowed the identification of ‘high scorers’ based on a calculated risk score determined by background characteristics and those reporting risky behaviours as ‘risky sleepers’. Semistructured telephone/online interviews took place from August to December 2022. Reflexive thematic analysis identified key themes.

**Results:**

Twenty-nine interviews were conducted with 28 families, with 14 ‘high scorers’ and 15 ‘risky sleepers’. The key themes were trustworthy sources, interpretation of risk and desperate times call for desperate measures. Caregivers of high-risk infants generally understood and followed safer sleep guidance but described rare occasions of compromised safety, usually due to routine disruptions. Those engaging in risky sleep practices cited factors such as sleep deprivation, past experiences, self-identity and personal interpretations of safer sleep advice as influential.

**Conclusions:**

Safer sleep messages are reaching families, but targeted support is needed to prevent rare but fatal incidents. Health professionals should consistently discuss co-sleeping using improved communication strategies to enhance trust. Co-created, accessible and bitesize resources should support parents in problem-solving and planning for safer sleep during disruption to routines.

**WHAT IS ALREADY KNOWN ON THIS TOPIC**

- Safer sleep advice has contributed to a significant decline in sudden and unexpected deaths of infants since 1990; however, the death rate remains high among families experiencing social and economic adversities.
- Recent data show that risky sleep environments are present in the majority of unexplained infant deaths in the UK and are a focus of national guidance.
- Previous research shows that knowledge of safer sleep messages does not guarantee safer sleep practices, and factors such as disruption to routines, sleep deprivation and didactic messaging can inhibit safe practices.

**WHAT THIS STUDY ADDS**

- We engaged a unique group of parents for interview, including those with background characteristics known to increase the risk of sudden unexpected deaths in infancy and those who reported engaging in practices that went against national safer sleep advice.
- Parents described risky scenarios as responses to real-life challenges, shaped by identity, past experiences and the perceived credibility of different sources of information.
- Competing interpretations of risks were influential in the decision making of this group, with some using online sources as justification for a particular practice.

**HOW THIS STUDY MIGHT AFFECT RESEARCH, PRACTICE OR POLICY**

- All caregivers should be offered a conversation with a health professional that acknowledges the possibility of rare but risky sleep situations, be given support to identify times when these situations might be more likely and be offered help with planning for safety during those times.
- Families who are considered ‘low risk’ may also benefit from a deeper understanding of how risks can change, with particular attention to bed-sharing and the added risk of alcohol to that environment.
- Approaches to safer sleep education should be co-designed, judgemental language and ‘scare tactics’ should be avoided and visually engaging media from trusted sources should be used.
- Risk reduction approaches that explain how to minimise risks to a baby in all sleep environments may afford more protection to infants than didactic approaches that include advice perceived as unrealistic by caregivers.

## Background

 Sudden unexpected death in infancy (SUDI) is a term used at the point of presentation to describe the death of an infant (under age 1) whose death was not considered a significant possibility in the preceding 24 hours.^1^ Following investigations, these deaths will be categorised into those that have a clear diagnosis (and are explained) and those that do not have a clear diagnosis and remain unexplained (also known as SIDS or sudden infant death syndrome).^2^ In 2022, the UK’s Office for National Statistics recorded 171 SUDI deaths that remained unexplained following review.^3^ SUDI is associated with premature birth, low birth weight, multiple births, larger families, admission to a neonatal unit and maternal smoking during pregnancy.^4^ Unsafe sleeping arrangements form part of the UK’s national guidance on SIDS risk reduction^5 6^ and were found by the National Child Mortality Database to be a modifiable risk factor in 72% of deaths.^7^ These unsafe arrangements included infants placed in the prone position for sleep, sleeping on a sofa or an armchair with an infant, and hazardous co-sleeping in the parental bed, namely bed-sharing where the adult has consumed alcohol or smokes, or sleeping with an infant under 12 weeks of age who was born preterm or with low birth weight.^4^

Here, co-sleeping is a term used to describe an adult and a baby sleeping together on the same surface, which could include sofas, chairs and adult beds. Bed-sharing is a form of co-sleeping, but specifically relates to an adult and a baby sleeping on an adult bed together. Both are distinct from room-sharing, where a baby and an adult sleep in the same room together but not necessarily on the same surface. Current UK guidance from The Lullaby Trust, the National Institute for Health and Care Excellence (NICE), the Royal College of Midwives and the National Health Service (NHS) states that rather than advising against co-sleeping, health professionals should educate families about the specific circumstances that make co-sleeping riskier, as described above. In the UK, health professionals should share with families the risks of unsafe sleeping arrangements and discuss how to reduce these.^5 6 8 9^ These messages often come from health professionals (midwives and health visitors), with wider influences from family members, peers and the media. Exploring whether or not these messages reach every parent, understanding how they then navigate safer sleep information and examining the influences on their decision-making around infant sleep safety can provide insights into how best to support families to follow safer sleep advice.^10^

Previous insights into the night-time care of infants have provided detailed accounts of how mothers navigate complex decisions without much support.^11^ They were guided by their own previous experiences and questioned the credibility of ‘didactic, unhelpful advice’ from health professionals regarding infant sleep position, co-sleeping, smoking, dummy use, feeding and disrupted routines. In 2020, the National Safeguarding Review Panel^12^ called for further research into behavioural insights of families with at-risk children to develop and deliver effective safer sleep messages and approaches. Several studies formed part of the response,^13 14^ including a mixed-methods study^15^ that used the COM-B (Capability, Opportunity, Motivation, Behaviour) model of behaviour change to identify modifications to practice involving the delivery of safer sleep messages to parents who have a social worker. The study found that motivational factors (eg, parental sleep deprivation and the desire to bond) were important, that the credibility of the messenger made a difference in the acceptability of the advice and that social pressures of good parenting may interfere with acknowledging and planning for ‘out of routine’ circumstances.

The current study explores infant sleep decision-making among families where targeted support may have the biggest impact: caregivers reporting unsafe sleep practices and those with infants at increased risk of SUDI. By exploring the views of these two priority groups, we hope to inform future efforts to support families in all scenarios, where the risk to the baby is already higher but also where following the advice is more challenging.

## Methods

### Objective

This qualitative study examined caregivers’ decision-making regarding infant sleep safety. Purposive sampling was used to select eligible participants based on the following criteria: caregivers of infants under 12 months, infants at increased risk of SUDI (‘high scorers’) or those who report risky sleep practices (‘risky sleepers’). Semistructured interviews explored their understanding of sleep safety and care practices.

### Recruitment

An online survey (August–December 2022), distributed via health clinics, children’s centres and social media, identified eligible participants and collected consent to be contacted for interview. A risk score developed in a previous study^16^ identified infants scoring 100+ (‘high scorers’) based on maternal age, sex, birth weight, neonatal unit admission, smoking, partner support and partner smoking status, if applicable. ‘Risky sleepers’ reported one or more hazardous sleep practices in the previous night’s sleep: placing an infant under 6 months old prone for sleep or co-sleeping with hazards present (co-sleeping with an infant after consuming alcohol or recreational drugs, with someone who smokes, with a preterm or low birthweight baby under 12 weeks old, or on a sofa).

### Data collection and analysis

Those who gave consent to be contacted for an interview and who met one or both criteria were sent a participant information sheet and were invited for an interview with a member of the research team (BL, A-AK or AP). All three researchers who conducted the interviews were female, all held PhDs in social science-related topics and all were trained and experienced in conducting qualitative research. Individuals aged <16 years, those who lacked the cognitive capacity to consent and those unable to complete an interview in English were not eligible to take part in the study. Participants were thanked with a £20 shopping voucher.

The semistructured interviews were conducted either by telephone or online and lasted approximately 1 hour. The interviews were structured around a topic guide (online supplemental appendix A) that was adapted based on each individual’s survey responses. The guide focused on (1) the infant’s sleep environment, asking questions about the position, location, any sleep deprivation and changes in routine; (2) sleep safety advice and sources of support, exploring participants’ thoughts surrounding safer sleep messaging, as well as the sources of their information and what further influenced their beliefs; and (3) their preferences for resources, that is, what formats the participants currently engaged in when accessing information related to infant safety, their preferences for accessibility and style, and their thoughts on trustworthiness.

Audio recordings were transcribed and analysed by three coders (AP, A-AK and BL) using reflexive thematic analysis,^17^ informed by a phenomenological approach,^18^ using NVivo software.^19^ An interpretative reflexive approach was used and allowed for theme development using both semantic and latent interpretations of the data.^20^

### Patient and public involvement

The Baby Sleep Project family advisory group comprises 15 caregivers of young infants with a variety of experiences, including multiple births, neonatal stays, socioeconomic deprivation, young age, social care involvement and homelessness. The group met regularly throughout the study design and data collection to guide the research team. The group gave guidance on the content and wording of the participant study documents, gave insights into what should be included in the topic guide and supported the team with ideas to maximise recruitment to the study. The study team followed UK Standards for Public Involvement guidance^21^ to make sure that the advisory group were clear about what was being asked of them, provided “you said we did”-style feedback after every meeting and ensured reciprocity via appropriate payment with bank transfer or with a shopping voucher. Contributions by the advisory group participants were also recognised via the study website (https://babysleepresearch.co.uk/family-involvement/).

### Ascertainment

Of the 3100 survey respondents, 75 (2.4%) were ‘high scorers’ (range 104–168, mean 129) and 41 agreed to be contacted for interview, with 14 completing the interview. Of 3025 lower-risk respondents, 69 reported hazardous sleep practices and 15 were interviewed, resulting in a total of 29 interviews, which ranged from 30 min to 1 hour. One participant was identified as a high scorer and also reported risky sleep practices; this participant was classified as a ‘high scorer’ for the purposes of analysis. Findings from both groups were analysed to identify central themes, supported by representative participant quotes in tables 2–4.

### Characteristics of the participants

Of the 29 participants, 4 were mothers under 25 years old, 8 had four or more children and 10 smoked during pregnancy. Thirteen were current smokers. Most participants (26 out of 29) had partner support, with nine partners also smoking. Nineteen infants were male, nine had low birth weight (<2.5 kg), nine were preterm, and eight had required admission to the neonatal unit. Further demographic information is shown in table 1.

**Table 1:** Participants’ demographic information

Role	n/N (%)
Biological mother	25/29 (86)
Biological father	1/29 (3)
Foster mother	3/29 (10)
Household income above £17 940 per year?	
Yes	23/29 (79)
No	6/29 (21)
Baby’s ethnicity	
White: English, Welsh, Scottish, Northern Irish or British	25/29 (86)
Irish	1/29 (3)
White and black Caribbean	1/29 (3)
White and South American	1/29 (3)
Other white background	1/29 (3)
Education level	
General Certificate of Secondary Education (GCSE) level or above	29/29 (100)
Involvement with social care services?	
Yes	4/29 (14)
No	25/29 (86)

### Infant age and SIDS awareness

Infants’ age ranged from birth to 12 months, with the age of those in the ‘high scorer’ group ranging from under 4 weeks old to up to 9 months old, with a median of 4.2 months. Babies in the ‘risky sleeper’ group ranged in age from under 4 weeks to up to 11 months old, with a median of 4.1 months. Age distribution, caregiver relationships and discussions about SIDS with health professionals were similar in both groups.

### Themes and subthemes

**Figure 1:**
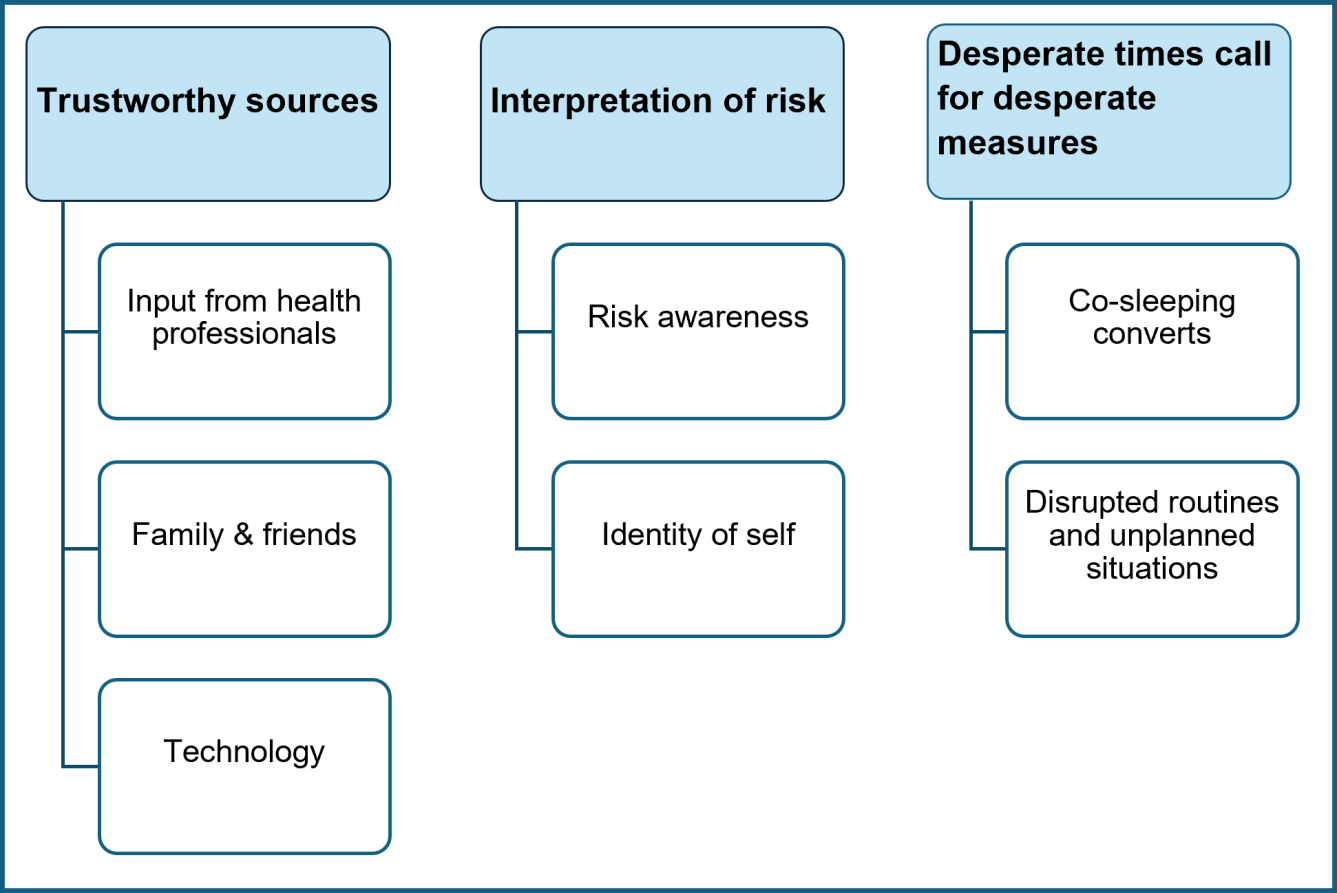
Themes and subthemes.

Three themes and seven subthemes resulted from the analysis. These are displayed in figure 1 and discussed thereafter with illustrative quotes from the participants (with anonymous ID indicating whether they were a high scorer or a risky sleeper and the age of the infant) as shown in tables2^4^.

**Table 2:** Illustrative quotes for theme 1: trustworthy sources

Subtheme title	Quote number	Quote
Input from health professionals	Q1	“We talked about bed sharing, and they told us the dos and don’ts. Basically you can’t really do it in case you roll over and suffocate him.” (HS8, infant 13–16 weeks old)
	Q2	“My midwife told me not to [bed-] share if you’ve had a drink, if you’re a smoker, make sure there’s nothing on the bed if she’s sleeping on the bed, make sure she’s safe. There’s an increased risk of SIDS with co-sleeping.” (HS10, infant 5–8 weeks old)
	Q3	“They [health professionals] were saying “if you do this he will die” … I would understand if they walked in and his cot was covered in cot bumpers and a big cover it and it had toys everywhere, it’s not … And they still bang on about it to the point that it gets you that paranoid. The first two weeks of his life I was sat up watching him sleep.” (HS13, infant 29–32 weeks old)
	Q4	“In our NCT [National Childbirth Trust] class they did say to look up the Lullaby Trust guidance because you probably will end up co-sleeping.” (RS13, infant 25–28 weeks old)
	Q5	“…even in this country people get told they’re putting their baby at risk for co-sleeping in the night which really then makes you not want to speak about it.” (RS1, infant 13–16 weeks old)
Family and friends	Q6	“We have pillows and we barricade the sides with pillows, all down the sides and across the top and bottom … We got it [this advice] from my partner’s mother, it’s what she used to do.” (HS10, infant 5–8 weeks old)
	Q7	“It’s down to you to decide what information you decide is for you, because if anything happens, it’s on you.” (HS13, infant 29–32 weeks old)
	Q8	“It was my first time being a mum … so during that time I was enquiring a lot from friends, family, and also a lot of browsing from the internet.” (HS4, infant 33–36 weeks old)
	Q9	“It’s the Lullaby Trust. I think that’s been my go to for most things safe sleep, but I have done a lot of research into safe sleep.” (RS2, infant 25–28 weeks old)
	Q10	“I never really asked anyone else about safe [sleep], I never asked my mum because her advice is massively outdated.” (RS1, infant 13–16 weeks old)
Technology	Q11	“With [short form videos], it’s a bit shorter for people, you just get the information that’s in a quicker and simpler way, whereas they’re not going to be overloaded and they haven’t got to sit there for half an hour and read five paragraphs.” (HS11, infant 0–4 weeks old)
	Q12	“You know when they have a tick next to the account? They’re a real business or celebrity or something.” (HS14, infant 13–16 weeks old)
	Q13	“I would say I get 90% of my information from [social media], because it’s in little bite sized chunks that my brain is capable at the moment of digesting … I don’t know what they’re … they call themselves sleep consultants. …I don’t think they’re clinically trained, but they just have stuff about what normal infant sleep looks like, and I guess to make you feel better that it’s not just you with a baby that wakes up every hour.” (RS13, infant 25–28 weeks old)
	Q14	I guess some of the [social media] accounts that I follow are about sleep. I think it’s just they do give the information but I don’t really look … I haven’t looked into them, but I use my own intuition. If it doesn’t seem right I just won’t try what they say.” (RS6, infant 13–16 weeks old)
	Q15	“I try to avoid social media, because there’s so many people out there on websites […] claiming to be sleep consultants and sleep trainers and there’s never any proof of any qualification or any training.” (RS14, infant 9–12 weeks old)
	Q16	“We share a lot of the material in my group. I like them, I feel like they know what they’re talking about … I’ve had a look and they’re all actual health professionals … They have done the research.” (HS13, infant 29–32 weeks old)
	Q17	“They’ve got a little co-sleeping video on there which was quite useful. It was quite simple, easy to understand … I sent that video to my partner and it was just an easy information visual guide.” (RS2, infant 25–28 weeks old)

Quotes are followed by participant ID, where HS is high scorer and RS is risky sleeper, and the age of the infant.

SIDS, sudden infant death syndrome.

**Table 3:** Illustrative quotes for theme 2: interpretation of risk

Subtheme title	Quote number	Quote
Risk awareness	Q18	“I leave her alone in the bed, make sure she’s safe, because I am a smoker.” (HS10, infant 5–8 weeks old)
	Q19	“If I’m tired I will just go into the bed with her, I know that it’s safer [than sofa-sharing], and if I nod off it doesn’t matter.” (RS13, infant 25–28 weeks old)
	Q20	“Sometimes we’ve put him just on the sofa by himself and then cushions on the floor so if he did fall it lessens the impact … for his little daytime naps he’s with us on the sofa.” (RS12, infant 0–4 weeks old)
	Q21	“I know she’s smaller … not quite as developed, but we co-sleep, I think purely from a point of that I need to sleep … I just feel broken and I can’t keep doing this.” (RS15, infant 5–8 weeks old)
Identity of self	Q22	“I’m a nursery nurse practitioner so I learnt from the course when I studied it years ago.” (HS11, infant 0–4 weeks old)
	Q23	“One child I have fostered his dad fell asleep with him on his chest while they were in bed and he rolled and got harmed as a result of that … so it’s more prevalent for us [as foster parents] when we see the effects that co-sleeping can do.” (HS5, infant 17–20 weeks old)
	Q24	“I’ve always been careful with that [co-sleeping]. I follow rules because it’s … I’m not willing to risk something happening to them.” (HS13, infant 29–32 weeks old)
	Q25	“She’s actually baby number four, and it’s ended up happening with all of them.” (RS15, infant 5–8 weeks old)
	Q26	“I think if I was a first- time mum then I might be a little bit concerned, but I think when you’re a second time mum you know what you’re doing.” (RS5, infant 25–28 weeks old)
	Q27	“I think a lot of rules and recommendations you’ve got to go with your own instincts … it’s following them as much as you possibly can, but be adaptable.” (RS7, infant 21–24 weeks old)
	Q28	“I wish we could follow them. I just really struggle because he is up so frequently in the night, so it would mean not drinking at all, which I did for the nine months of pregnancy and I felt like I’d had my life back a little more now.” (RS2, infant 25–28 weeks old)

Quotes are followed by participant ID, where HS is high scorer and RS is risky sleeper, and the age of the infant.

**Table 4:** Illustrative quotes for theme 3: desperate times call for desperate measures

Subtheme title	Quote number	Quote
Co-sleeping converts	Q29	“I was dead set against her sleeping in our bed … whereas now I trust myself with her in my bed, and I think with her being a second child I’m a lot more laid back, because you can base what you’re doing now on past experiences.” (RS5, infant 25–28 weeks old)
	Q30	“He was waking up every hour, and the way that we coped is that we got him in bed with me and he’d feed whenever he wanted to.” (RS13, infant 25–28 weeks old)
	Q31	“I know there’s more risks if they’re co-sleeping. I do quite often wake up thinking I’ve smothered her, or … yeah, so I know there are the extra risks …, but I can’t listen to her screaming in a crib.” (RS15, infant 5–8 weeks old)
	Q32	“Safe sleep, it’s okay in theory, but in practice it … well for us it just hasn’t worked.” (RS3, infant 45–48 weeks old)
	Q33	“Finding the Lullaby Trust was a game changer, because it went from having absolutely no sleep, to having some sleep with him next to you, so that was really helpful and just gave me the confidence to do it.” (RS13, infant 25–28 weeks old)
Disrupted routines and unplanned situations	Q34	“We’ve got a campervan and he’s spent a lot of nights in there already … he co-sleeps with us, it’s a mattress on the floor essentially, on the floor of the van.” (RS2, infant 29–32 weeks old)
	Q35	“They gave us a travel cot … but it didn’t work. So I shared a bed just with him, so I could keep him in the middle of the bed.” (RS8, infant 25–28 weeks old)

Quotes are followed by participant ID, where RS is risky sleeper, and the age of the infant.

#### Trustworthy sources (quotes 1-17)

##### Input from health professionals

National health guidelines recommend that babies sleep in a clear, flat sleep space in the same room as the adult for the first 6 months. They acknowledge that some parents co-sleep and suggest that babies sleep on their back, on a firm mattress, with no pillows or other children/pets. Bed-sharing is discouraged for low birthweight and premature babies or where adults smoke, drink alcohol or use drugs. All 29 participants described receiving safer sleep advice from health professionals, with varying guidance on co-sleeping, ranging from discouragement to risk minimisation in bed-sharing situations (table 2).

Within the high-scoring group, co-sleeping/bed-sharing messages were interpreted as a warning about suffocation as the mechanism of risk, to make sense of the advice (Q1). The high scorers also described more indepth conversations regarding SIDS and sleep safety when compared with the risky sleepers. The tone of these conversations felt more explicit and personal to them (Q2). Health professionals are encouraged to deliver messages of risk without scaring the parents while also balancing the need to encourage parental confidence and autonomy with their own duty of care; however, some parents described experiences where they felt that fear tactics had been used (Q3).

Some of the risky sleepers described their experience of health professional input as primarily signposting to resources about co-sleeping (Q4). Participants discussed difficulties in trying to find a balance between trustworthy information, understanding the risk and being an ‘expert by experience’. Some parents felt anxious about co-sleeping for fear of repercussions from their health professional (Q5).

##### Family and friends

Parents described balancing information from healthcare professionals with advice from friends and wider family members. Some of the high scorers reported seeking reassurance from experienced mothers and weighing this up against advice from health professionals. Where participants reported health professionals as rarely available, advice from family members and/or friends was favoured.

One participant, for example, was hesitant to co-sleep after receiving advice from her health visitor. However, as her baby struggled to settle due to reflux, she followed her mother-in-law’s advice about co-sleeping in an adult bed, using pillows as a barrier, despite this being against official guidance (Q6). Another participant gathered knowledge from multiple sources, including friends, but weighed this up against their own judgements (Q7).

For other high scorers (particularly younger, first-time parents), peer advice played a pivotal role in shaping their decision-making (Q8). The risky sleepers preferred to conduct their own research over generational or peer advice, acknowledging that advice has changed over time (Q9, Q10).

##### Technology

Majority of the participants from both groups stated that technology played a significant role in shaping their understanding of infant sleep. Sleep-tracking apps, social media groups and influencers’ videos were some of the ways parents shared questions and received information regarding infant sleep. These resources were a convenient resource for those participants. Within the high-scoring group, the younger parents, in particular, preferred accessing social media platforms for advice instead of the UK’s NHS website. The accessible, bitesize information from sources they felt to be trustworthy was attractive to them (Q11, 12). Similarly, many of the risky sleepers followed ‘sleep experts’ on social media who shared advice and sometimes promoted sleep products. However, trust in these experts varied (Q13, Q14, Q15).

Trust acted as a motivation for following guidance. What made a source ‘trustworthy’ differed between participants; one person described how social media accounts that were ‘verified’ (had a blue tick next to their name) were trustworthy (Q12), while another explained how they used their intuition to evaluate if the information was legitimate (Q14). Many participants named the NHS as a trustworthy source of guidance, while others were wary of it and felt it was either outdated or inaccessible (too lengthy and text-heavy) compared with the attractive, bitesize information shared via social media. The Lullaby Trust was a source of information regularly accessed by most participants from both groups that successfully bridged the gap between trustworthy health guidance and bitesize information (Q16, Q17).

### Interpretation of risk (quotes 18–28)

#### Risk awareness

Although most participants from both groups were aware of safer sleep messages, there were varying interpretations of risk. For example, one high-scoring participant who understood the risks associated with smoking and co-sleeping chose to leave their baby alone in an adult bed, despite the increased risk, because they felt this was safer than bed-sharing (Q18). Some of the risky sleepers acted against national guidance by sharing an adult duvet in their adult bed with their baby because they felt it was safe to do so since they were ‘not a smoker’, while others in this group felt that certain sleep safety messages did not apply to them if their baby had not spent time on a neonatal unit (table 3).

Some parents understood the dangers of sofa-sharing while sleeping and avoided this by bed-sharing instead, despite having consumed more than two units of alcohol or having smoked cigarettes (Q19). Conversely, other participants viewed sofa-sharing (with known risks) as a ‘last resort’ out of fear of the risks associated with bed-sharing (Q20). Others found themselves unable to follow the guidance due to sleep deprivation (Q21).

#### Identity of self

How participants saw themselves affected the decisions they made around their baby’s sleep; for example, one participant who smoked did not see themselves as a ‘smoker’ as they felt their smoking level was low. Some participants described their work experience or identity as a professional in childcare as the basis for their safer sleep knowledge, and their identity within childcare roles influenced their decisions (Q22). Identity as a foster carer strongly influenced one participant’s decision to not co-sleep (Q23).

Within both groups, participants with other children often referred to their identities as experienced parents as the basis for safer sleep practices. The high scorers were likely to follow national safer sleep guidance, with several mentioning doing so with their other children (Q24), whereas for the risky sleepers ‘being a co-sleeper’ with their previous children seemed to mean they were more confident to take risks again with their current baby. Having co-slept previously was one of several reasons given by some participants when asked why they co-sleep with their baby in a hazardous environment (Q25).

According to some participants, health professionals were less engaged when the parent had existing children, assuming that the parents would already know the advice, which in turn led to the parent feeling more confident to engage in hazardous co-sleeping (Q26). Motherly instinct was also given as a justification by other participants who were aware of the risks (Q27). One of the five risky sleeper participants who had consumed more than two units of alcohol and co-slept the night before described how her identity had changed from prepregnancy to postbirth and how this had meant she now struggled to follow certain ‘rules’ (Q28).

### Desperate times call for desperate measures (quotes 29–35)

#### Co-sleeping converts

Some ‘risky sleepers’ described how they had become ‘co-sleeping converts’, having had no intention to do so during pregnancy (Q29). Understanding the risks of co-sleeping and choosing to do it anyway was a common finding among the majority of risky sleepers. Parents who found themselves in this situation often described desperate experiences of sleep deprivation where they had ‘tried everything else’ and started to co-sleep ‘as a last resort’ (Q30, Q31, Q32). These parents felt they had no other option but to co-sleep, and several of them had felt anxious discussing co-sleeping with their health visitor for fear of repercussions. The Lullaby Trust’s specific guidelines on how to co-sleep with the baby more safely (how to arrange the bedding, etc) were appreciated by several parents as a reassuring and realistic resource (Q33). It is clear that even where risks are present and where following the guidelines would involve avoiding co-sleeping, some parents found it useful to have practical information about decreasing the risks to the baby in an adult bed (table 4).

#### Disrupted routines and unplanned situations

Disruptions to the usual routine are known to impact on safe infant sleep.^4^ Certainly, in the current study*,* changes to routine had led some parents to take risks, for example, on a holiday in an accommodation with no designated sleep spaces for infants (Q34). Unplanned situations such as illness or other disruptions such as staying away from home also increased the likelihood of parents co-sleeping in hazardous circumstances (Q35).

## Discussion

All participants demonstrated knowledge of safer sleep guidance from health professionals before and after birth. The advice regarding co-sleeping given to parents by health professionals ranged from total discouragement in any circumstances (which is contrary to the UK national guidance as co-sleeping is advised against only where specific risks are present) to guidance on how to reduce the risks in a bed-sharing situation. Most high scorers placed their baby in a separate sleep space, viewing bed-sharing as risky.

Risky sleepers generally reported feeling ‘well educated’ about safer sleep, yet were not following national guidance consistently, whereas the high scorers sometimes preferred peer sources but reported safer sleep more consistently. This fits with our understanding of how infant vulnerabilities impact on the likelihood of SIDS occurring and also with how we understand that knowledge and awareness of SIDS risk reduction messages are only one small part of the effectiveness of interventions. It may be that even where parents express confidence in their own safer sleep knowledge, they still need support with making plans for following the guidance more consistently, especially during times when their routines are disrupted.

Some participants described limited contact with their health visitor and reported searching online for answers to their questions. Younger participants felt as though they had nowhere else to go. Official health guidance from the NHS was often felt to be ‘too wordy’ or ‘full of jargon’, whereas social media platforms were more accessible. Subsequently, social media influencers contributed to the parents’ belief that ‘what works for others will work for me’, an insight reiterated within existing research where young people judged the credibility of sources by ‘instinct and appearance’.^22^ The ‘bitesize’ information on such platforms, using videos and with minimal text, was how most participants in this study accessed advice. Recent studies have focused on imagery, showing that significant proportions of infant sleep images are contrary to safer sleep guidance,^23^,^25^ highlighting the need for trusted and credible sources of information on infant safety to engage parents via social media and to ensure accessibility of evidence-based information.

Although most participants knew safer sleep guidelines, some still engaged in risky practices, a finding echoed in other qualitative studies, showing the gap between giving advice and being able to follow it.^26^,^28^ Feeling that their baby was a ‘low risk baby’ may mean that carers have more tolerance to risky practices, with some in the current study using online sources to justify unsafe habits. Others described unintentionally increasing the risk of SIDS out of desperation for sleep; again, sleep deprivation is a commonly cited reason for difficulty following safer sleep advice.^29^ Current practical advice on how to reduce risks during intentional or unintentional bed-sharing was found to be useful to some even though they were bed-sharing with hazards present. This is similar to ‘risk minimisation’ approaches taken in some countries, including Australia.^30^,^33^ Future research should focus on how this approach influences practice, with close monitoring of deaths and the associated risk factors.

High scorers described the need to weigh up national guidance against generational advice from family members, recognising why their baby was at increased risk and how their behaviour could increase that risk. The influence of generational advice on infant care practices is well documented,^34^ especially for younger parents, meaning that risk reduction advice should aim to engage wider family and peers as often as possible. When it came to co-sleeping, many understood hazardous co-sleeping dangers; however, for those who co-slept the night before, this felt like the only option and information about reducing the risks in this environment was not available to them. This has implications for health professionals and the sharing of resources, such as The Lullaby Trust’s co-sleeping guidelines.^5^

Unplanned situations and disruptions to usual routines meant that some participants were more likely to take risks, a finding echoed in other studies.^12^ Resources to help plan for these disruptions could therefore potentially support some families to follow safer sleep advice more consistently. Related to the current study is the development of an online risk assessment and safety planning tool to support families in understanding their infant’s risk of SIDS and suggest ways to plan for safer sleep, including when usual routine is disrupted.^35^

Similar to other studies, we found that the framing of advice given by health professionals has implications for a caregiver’s interpretation of the associated risks.^36^ High scorers often received strong discouragement against co-sleeping, while risky sleepers were advised on safer co-sleeping rather than complete avoidance. Socioeconomic factors, including poverty and poor housing, influence parenting choices and risk of SIDS.^37^ It was a positive finding that in this study the high scorers appeared to have been receiving regular input from a health professional. The risky sleepers reported experiencing infrequent health professional input and did not report receiving the more explicit, personalised messages about sleep safety. Given that both groups occasionally found themselves in risky scenarios, support with planning for risk reduction during disruptions to routine should be available to all parents, regardless of background risk status.

Self-identity and risk perception influence risk, leading to low-risk babies sometimes being placed in high-risk situations. A Lullaby Trust survey found that 9 in 10 parents co-sleep at some point, yet fewer than half had been given specific advice about reducing the risk of SIDS in co-sleeping situations.^38^ Providing safer sleep advice requires finding a balance between providing explanations of the risk and the rationale and providing reassurance.^15^ Some participants in this study discussed wanting to ask their health professional about co-sleeping but felt too worried about doing so for fear of judgement. Future evidence-based resources that share safer sleep advice should therefore be coproduced with family members, including families with high-scoring infants and those who are vulnerable to disrupted routines.

### Strengths and limitations

The study successfully explored decision-making around infants’ sleep environments, encompassing those with infants at risk and those who reported not following safer sleep advice. Given recent public health efforts to target those most at risk, this study provides unique insights from a priority group of families. Social desirability bias may have influenced the reporting, although efforts were made to mitigate this by ensuring confidentiality and emphasising the value of honest accounts. We were only able to conduct interviews in English, limiting our ability to investigate decision-making for families who speak other languages. Future research should aim to address this gap in the literature in the UK. Interviews were conducted over the telephone or online, which excluded those without access to either option. Again, future research should aim to include face-to-face interviews where possible to make the research more inclusive. Like all qualitative research, the findings are limited in generalisability, and future quantitative studies should seek to confirm, refine or refute our findings.

## Conclusions

Given that infants with known risk factors suffer disproportionately higher mortality, our evidence may indicate that acknowledging rare but risky situations as possible, offering support in recognising when these situations are more likely and providing support in planning for safety during times of disruption may be particularly important.

For risky sleepers, self-perception and external influences affected risk interpretation, and it was found that traditional didactic messages or scare tactics were ineffective. External advice from family, friends and social media influenced risky decisions, and where health professionals are seen as credible sources they can mitigate risks through discussion with the family. Tailored, practical resources to support effective conversations with families, coproduced with caregivers, may help address their needs and improve the communication of safer sleep advice.

For the last 30 years, infant lives have been saved by evidence-based advice on how to reduce the risk of SIDS. Risk reduction approaches in public health acknowledge that less-than-ideal situations are a normal part of life and provide pragmatic solutions to complex scenarios. Approaches that used fear as a motivator or ‘scared straight’ tactics seemed not to work for this group. Similarly, efforts to eliminate all risks from an infant’s sleep environment may backfire, as seen in this study where families tried to navigate rigid, or idealised, advice and ultimately failed to follow it. Risk reduction approaches that support caregivers’ understanding of how and why safer sleep advice protects infants and support with planning for safety during times of disruption may help further reduce deaths.

## Supplementary material

10.1136/bmjpo-2025-003620 online supplemental file 1

## Data Availability

Data are available in a public, open access repository.
