# Supplementary material for: “Okay in theory”: a qualitative study of safer sleep advice in families with infants at risk and families reporting risky sleep practices
Source: BMJ Paediatr Open. 2025 Jul 24;9(1):e003620. doi: 10.1136/bmjpo-2025-003620 (PMC12306268; doi:10.1136/bmjpo-2025-003620)
Supplement: online supplemental file 1 [file bmjpo-9-1-s001.docx]

### Appendix A: Interview Topic Guide

Baby Sleep Project Topic Guide for Interviews

Introduction

- Thank participant for meeting
- Check participant is still happy to be involved with the research
- Explain recording device / confidentiality
- Explain purpose of the interview and that participant does not have to talk about anything they feel uncomfortable with and can stop at any time for any reason
- Check consent form is signed

**Interview part 1: The sleep environment**

Topics to include:

- Sleep last night – how did you all sleep last night?
- Position – what position does your baby sleep?
- Locations – where does your baby sleep?
- Dummies – does your baby use a dummy? When you woke up was the dummy still in her mouth?
- Swaddling – what does your baby wear for sleep? What kind of coverings keep them warm?
- Products – Some parents buy things to help their babies sleep, do you use anything like that? Eg pod or next or hammock
- Other people’s influence – Where do you get your information from about baby sleep?
- Sleep disruption / wakefulness / sleep deprivation – have you had any times where sleep has been lacking for you? What was that like? What did you do to cope?
- Changes in routine / disruption that impacts on infant care – have you had any times where the normal routine hasn’t happened? What was that like? Can you describe what happened?

**Interview part 2: Advice and sources of support**

Topics to include:

- Role in decision making as main carer or supporter – who makes the decisions about how to look after the baby?
- Advice heard, thoughts/opinions/beliefs about that – what things have you heard about infant sleep? What did you think of those things? Did you think they were true or not? Why?
- Other people’s influence – who else helps you with advice for how to look after the baby?
- Sources of information (including health professionals, peers, family, Internet, social media etc) What about other ways of getting advice – have you looked up things about sleep on the internet? Or used social media to find out about infant sleep?

**Interview part 3: Preferences for resources**

Topics to include:

- Formats engaged – social media, video, websites, apps, paper – if we were going to come up with information to help families with their baby’s sleep where should we have it? Should it be on a website? Should it be in a video? What kind of videos do you watch? What about animations? Would you use an app that was about baby’s sleep?
- Topics they would like info on – safety, sleep deprivation, products, etc
- What makes something trustworthy? Can you think of any baby related information that you’ve seen that you thought was good? Why did you think it was true?
