# Supplementary material for: “Okay in theory”: a qualitative study of safer sleep advice in families with infants at risk and families reporting risky sleep practices
Source: BMJ Paediatr Open. 2025 Jul 24;9(1):e003620. doi: 10.1136/bmjpo-2025-003620 (PMC12306268; doi:10.1136/bmjpo-2025-003620)
Supplement: Supplementary file 2 [file bmjpo-2025-003620.reviewer_comments.pdf]

PEER REVIEW HISTORY

BMJ Paediatrics Open publishes all reviews undertaken for accepted manuscripts. Reviewers are asked to complete a checklist review form and are provided with free text boxes to elaborate on their assessment. These free text comments are reproduced below.

ARTICLE DETAILS

**TITLE (PROVISIONAL)**	"Okay in theory": A qualitative study of safer sleep advice in families with infants at risk and families reporting risky sleep practices.
**AUTHORS**	Lambert, Becky; Keegan, Alice-Amber; Ingram, Jenny; Blair, Peter; Fleming, Peter; Pease, Anna

VERSION 1 - REVIEW

**REVIEWER NAME**	***Joanna Garstang***
**REVIEWER AFFILIATION**	University of Birmingham College of Medical and Dental Sciences, School of Nursing and Midwifery
**REVIEWER CONFLICT OF INTEREST**	N/A
**AI DISCLOSURE**	No
**DATE REVIEW RETURNED**	28-May-2025

**GENERAL COMMENTS**	Peer review<br>This is an interesting paper that merits publication. It would be helpful to have a greater understanding of the differences and similarities between high scoring and risky parents decisions; this would lead to clearer recommendations for messaging with risky parents.<br>I have some minor comments.<br>Abstract<br>Methods – describe in chronological order.<br>Background<br>It may be better to define SUDI before introducing the concept of unexplained SUDI.<br>129 unexplained SUDI is probably an under-estimate due to delays in finalising case reviews and undersells the scale of the problem. NCMD data release or ONS sudden infant deaths may be more accurate<br>The information on current safe sleep guidance should include that although co-sleeping is not recommended against in general, it clearly states when parents should not co-sleep. It would help an international audience to state these.<br>There should be a definition of co-sleeping so that it is not confused with room sharing or anything else.<br>Methods<br>How was the online survey distributed?<br>What was the maximum score?<br>Results<br>'Eligible based on risk -score' maybe rephrase as 'high scorers'<br>How many interviewees were both risky and high scorers?<br>'Four were biological mothers under 25' suggest this is rephrase as it sounds as if only 4 biological mothers participated.<br>Infant ages ranged birth – 12 months – this was the eligibility criteria. What was the mean or median age for each group?

	Q4 – Is a NCT leader a healthcare professional? NCT is a voluntary group so needs some clarification. Non-UK readers will have not know NCT<br>Q6 – this needs some further context as the quote describes hazardous co-sleeping with all the pillows<br>Q9 – they may have done a lot of safe – sleep research but as they are risky this implies that they are not following it – this needs some discussion<br>Co-sleeping converts. The Lullaby Trust guidance is to avoid co-sleeping with other hazards (drugs/smoking/alcohol/ LBW etc). How does this advice then reassure a risky co-sleeper? In effect, they are ignoring the guidance.<br>Discussion<br>'total discouragement (contrary to national guidance)' please clarify how this contrary to national guidance ie co-sleeping not discouraged for low risk infants.<br>'High scorers often received strong discouragement against co-sleeping, while risky sleepers were advised on safer co-sleeping…'<br>This needs some further discussion. The risky sleepers may have been advised on safe co-sleeping but are not following this advice (if they did they wouldn't be risky). It would be helpful to have a greater understanding on the differences (if there are any) between risky and high scorers?<br>The LT survey reporting 9/10 parents co-sleep – presumably that is at some point rather than regularly? Please clarify.<br>Conclusion<br>'families with infants at increased risk are overrepresented in the deaths…' suggest rephrase – of course deaths happen more often in infants at increased risk of death

**REVIEWER NAME**	***Nidia Milena Villamil Osorio***
**REVIEWER AFFILIATION**	None disclosed
**REVIEWER CONFLICT OF INTEREST**	N/A
**AI DISCLOSURE**	No
**DATE REVIEW RETURNED**	01-Jun-2025

**GENERAL COMMENTS**	1. Background: ...These unsafe arrangements included infants placed in the prone position for sleep, sleeping on a sofa or an armchair with an infant, and hazardous co-sleeping<br>in the parental bed, namely bed-sharing where the adult has consumed alcohol, smokes, or with an infant under 12 weeks of age who was born preterm or low birthweight.<br>¿Is it the same bibliography, number 1?, Please write down the reference<br><br>2. Please add more detailed information about The Lullaby Trust's guidelines and national guidance in the Background, as readers in other countries are not familiar with this information.<br><br>I congratulate the authors for this great work, which is of great relevance to public health.

VERSION 1 – AUTHOR RESPONSE

Comment	Response e.g., “Page 4, Paragraph 2: Updated SUDI definition; Reference #5 added for NCMD 2022 data”
Definitions & Context<br>• Define SUDI (and, if used, SIDS) before mentioning “unexplained SUDI.”	
Update or qualify the figure “129 unexplained SUDI in 2020.” If more recent NCMD/ONS data exist, replace or note underestimation due to case-review delays. Cite the exact source.	
In Background, list unsafe sleep practices (prone position, sofa/armchair, hazardous co-sleeping). Provide a precise reference (NCMD, NICE or equivalent). Please add more detailed information about The Lullaby Trust’s guidelines and national guidance in the Background, as readers in other countries are not familiar with this information.<br><br>Background: ...These unsafe arrangements included infants placed in the prone position for sleep, sleeping on a sofa or an armchair with an infant, and hazardous co-sleeping<br>in the parental bed, namely bed-sharing where the adult has consumed alcohol, smokes, or with an infant under 12 weeks of age who was born preterm or low birthweight.<br>¿Is it the same bibliography, number 1?, Please write down the reference	
Safe-Sleep Guidelines & “Co-Sleeping”<br>• Add a brief paragraph on The Lullaby Trust’s and NHS/NICE guidelines: when co-sleeping is permitted (healthy term infants, non-smoking, sober parents) and when it’s strongly discouraged (smoking, alcohol, drugs, infant <12 weeks, low birthweight or preterm).	
Define “co-sleeping” (bed-sharing on the same surface) and distinguish from “room-sharing” (infant in separate cot). For context, mention AAP recommendations if desired but keep focus on UK guidelines.	
Abstract<br>• Reorder Methods chronologically: (1) semi-structured interviews design, (2) online survey	

distribution (Aug–Dec 2022), (3) thematic analysis with NVivo. Abstract<br>Methods – describe in chronological order.	
State total number of interviewed families and break down how many were “high-risk” versus “risky-practice.”	
Methods<br>• Specify how the online survey was distributed (e.g., via clinics, parent groups, social media).	
Describe the risk-score calculation: list factors, maximum possible score, and threshold for “high risk.”	
Clarify eligibility (infants age 0–12 months) and sampling (e.g., purposive or stratified).	
If any participants fell into both “high-risk” and “risky-practice” categories, state how many and how they were classified in analysis.	
Results<br>• Replace “Eligible based on risk-score” with “High scorers.”	
Report the number of participants overlapping both categories.	
Rephrase “Four were biological mothers under 25” to “Four mothers were under 25 years old.”	
Provide mean or median infant age (with range or interquartile range) for each group.	
Define “NCT leader” (National Childbirth Trust volunteer, not a health professional).	
For Q6, note that pillows-as-barriers reflect family-devised risk-reduction, not official guidance.	
In Q9, briefly address that knowing safe-sleep research doesn’t guarantee adherence.	
Discussion<br>• Explain “total discouragement (contrary to national guidance)” by clarifying that UK guidelines do not universally forbid co-sleeping—only under specific risks.	
Expand on why “risky-practice” families persist despite “safer co-sleeping” advice (e.g., space constraints, cultural beliefs). ‘High scorers often	

received strong discouragement against co-sleeping, while risky sleepers were advised on safer co-sleeping…' This needs some further discussion. The risky sleepers may have been advised on safe co-sleeping but are not following this advice (if they did they wouldn't be risky). It would be helpful to have a greater understanding on the differences (if there are any) between risky and high scorers?	
Clarify whether "9 of 10 parents co-sleep" refers to any instance or routine practice; cite the exact Lullaby Trust report. The LT survey reporting 9/10 parents co-sleep – presumably that is at some point rather than regularly? Please clarify.	
Acknowledge limitations: potential social-desirability bias, English-only interviews, phone-based data collection.	
Conclusions<br>• Rephrase "families with infants at increased risk are overrepresented in deaths" to "Infants with known risk factors suffer disproportionately higher mortality." Conclusion<br>'families with infants at increased risk are overrepresented in the deaths…' suggest rephrase – of course deaths happen more often in infants at increased risk of death	
Emphasize the need for tailored, practical resources that reflect family contexts.	
References<br>• Ensure up-to-date SUDI figures from NCMD or ONS (with year) and full citation for The Lullaby Trust's safe-sleep guidelines.	
Cite the source for "unsafe sleep arrangements" (Reviewer 2's request).	
Confirm all UK guideline URLs and format references in Vancouver style, per BMJ requirements.	
Style & Formatting<br>• Maintain consistent terminology ("safe sleep," "co-sleeping," "room-sharing").	

Check that all tables/figures follow BMJ Paediatrics Open formatting (titles, legends, defined abbreviations).	
Review grammar: ensure correct tenses, subject-verb agreement, and concise phrasing.	
Background<br>It may be better to define SUDI before introducing the concept of unexplained SUDI.	
129 unexplained SUDI is probably an under-estimate due to delays in finalising case reviews and undersells the scale of the problem. NCMD data release or ONS sudden infant deaths may be more accurate	
The information on current safe sleep guidance should include that although co-sleeping is not recommended against in general, it clearly states when parents should not co-sleep. It would help an international audience to state these.	
There should be a definition of co-sleeping so that it is not confused with room sharing or anything else.	
Methods<br>How was the online survey distributed?	
What was the maximum score?	
Results<br>‘Eligible based on risk -score’ maybe rephrase as ‘high scorers’	
How many interviewees were both risky and high scorers?	
‘Four were biological mothers under 25’ suggest this is rephrase as it sounds as if only 4 biological mothers participated.	
Infant ages ranged birth – 12 months – this was the eligibility criteria. What was the mean or median age for each group?	
Q4 – Is a NCT leader a healthcare professional? NCT is a voluntary group so	

needs some clarification. Non-UK readers will have not know NCT	
Q6 – this needs some further context as the quote describes hazardous co-sleeping with all the pillows	
Q9 – they may have done a lot of safe – sleep research but as they are risky this implies that they are not following it – this needs some discussion	
Co-sleeping converts. The Lullaby Trust guidance is to avoid co-sleeping with other hazards (drugs/smoking/alcohol/ LBW etc). How does this advice then reassure a risky co-sleeper? In effect, they are ignoring the guidance.	
Discussion<br>'total discouragement (contrary to national guidance)' please clarify how this contrary to national guidance ie co-sleeping not discouraged for low risk infants.	

VERSION 2 - REVIEW

**REVIEWER NAME**	***Joanna Garstang***
**REVIEWER AFFILIATION**	University of Birmingham College of Medical and Dental Sciences, School of Nursing and Midwifery
**REVIEWER CONFLICT OF INTEREST**	N/A
**AI DISCLOSURE**	No
**DATE REVIEW RETURNED**	28-Jun-2025

**GENERAL COMMENTS**	The authors have addressed all the comments well, and the paper is much improved. It is a useful addition to scientific literature

**REVIEWER NAME**	***Nidia Milena Villamil Osorio***
**REVIEWER AFFILIATION**	None disclosed
**REVIEWER CONFLICT OF INTEREST**	N/A
**AI DISCLOSURE**	No
**DATE REVIEW RETURNED**	01-Jul-2025

**GENERAL COMMENTS**	Congratulations
